# An AI-Driven Virtual Patient Platform (CBT Trainer) for Training Cognitive Behavioral Therapy Practitioners Against Competencies: Mixed Methods Pilot Study

**DOI:** 10.2196/84091

**Published:** 2026-03-06

**Authors:** Tianyu Terry Zhang, Rob Saunders, Stephen Pilling, Ciarán O'Driscoll

**Affiliations:** 1 Research Department of Clinical, Educational & Health Psychology, Centre for Outcomes Research and Effectiveness (CORE), University College London, 1-19 Torrington Place, London, WC1E 7HB, United Kingdom, 44 07398556250; 2 Camden and Islington NHS Foundation Trust, London, United Kingdom

**Keywords:** cognitive behavioral therapy, artificial intelligence, virtual patients, competence assessment, competency-based education, clinical training, psychological therapy training, simulation-based learning, mobile app, digital health

## Abstract

**Background:**

Cognitive behavioral therapy (CBT) training faces significant challenges, including supervised practice with diverse cases, inconsistent feedback, resource-intensive supervision, and difficulties standardizing competence assessment.

**Objective:**

This study evaluated the acceptability and feasibility of CBT Trainer (TTZ), the first virtual patient platform to provide real-time feedback aligned with established competence frameworks. The mobile app trains psychological practitioners using standardized artificial intelligence patient interactions and the evaluation of therapist responses against competence frameworks to enable structured skill development in a controlled, repeatable environment that complements traditional training methods.

**Methods:**

This mixed methods pilot study used a 2-stage approach. Stage 1 involved usability testing with 4 participants. Stage 2 included 59 participants from psychological practitioner training programs (a Low Intensity CBT Interventions Program and a Doctorate in Clinical Psychology) who engaged with the CBT Trainer voluntarily for over 1 month. Measures of impact included the System Usability Scale (SUS), platform naturalistic engagement, poststudy questionnaire on perceived competency development, comparative evaluation against traditional role-play, and qualitative feedback.

**Results:**

Participants engaged voluntarily with the platform for an average of 95.24 (SD 134.58; median 45.34, IQR 11.57–105.15) minutes of active role-play. Platform usability was rated as excellent (mean SUS 82.20, SD 12.93). Self-reported competence improvement improved most in assessment skills (96.7%) and information gathering (66.7%). When compared to traditional peer role-play exercises, participants rated CBT Trainer moderately favorably (mean 5.90/10, SD 1.94). Qualitative feedback highlighted strengths in competency-aligned feedback, on-demand accessibility, and a psychologically safe practice space.

**Conclusions:**

This pilot study provides evidence that an artificial intelligence–based patient simulation shows promise as a supplementary training tool for psychological therapists who use CBT in their practice, particularly regarding accessibility and immediate feedback. Future research should use randomized controlled designs with objective competence assessments.

## Introduction

### Overview

The training of mental health professionals represents a critical challenge in addressing the global mental health crisis. Within the National Health Service (NHS), there have been initiatives aimed at improving the quality of care and mental health outcomes by improving access to evidence-based psychological therapies [1]. This requires developing a skilled workforce. In addition, as demand for psychological services continues to rise, there is increasing pressure on training programs to develop competent practitioners efficiently and effectively. Technological innovations, particularly machine learning and natural language processing, offer promising new avenues for enhancing mental health training through virtual patient simulations with competency-based feedback, potentially addressing long-standing challenges in traditional approaches.

### Training Competent Therapists: The Role of Competence Frameworks

Competency-based education has emerged as a dominant paradigm in mental health care training, providing structured frameworks essential for skill acquisition, assessment, and professional development [2]. Across therapeutic modalities, the University College London (UCL) Center for Outcomes Research and Effectiveness (CORE) competence frameworks provide a comprehensive hierarchical model of competencies required for effective practice, distinguishing between generic therapeutic competencies, basic therapy-specific technique (eg, CBT [cognitive behavioral therapy]) competencies, and meta-competencies [3]. These frameworks are operationalized through validated competency scales, which translate professional standards into measurable assessment tools. For example, the Cognitive Therapy Scale-Revised (CTS-R) [4], the revised form of Cognitive Therapy Rating Scale (CTRS) [5], is one of the main treatment competence measurement tools used for CBT therapist accreditation internationally, comprising 12 distinct competency domains, including agenda setting, feedback, collaboration, pacing, and interpersonal effectiveness [6]. Competence frameworks and scales like these serve multiple functions, such as providing structured developmental pathways [7,8], facilitating formative assessment [9], supporting summative evaluation [10], and promoting training consistency [11].

Nevertheless, implementing competency-based training and evaluation faces considerable practical and methodological challenges. First, the reliability of competence assessment remains inconsistent; although tools such as the CTS-R can achieve high interrater reliability under controlled conditions, such as when groups of raters work together, independent ratings often show poor-to-moderate agreement [3,10,12,13]. Second, resource limitations create substantial barriers to effective competence development. This includes demands on clinical supervisors, limited access for trainees to diverse clinical presentations, reliance on time-demanding assessment methods, and resource constraints affecting feedback quality [8,14]. Third, ethical tensions arise as trainees must develop competence “on the job,” with a need to ensure patient safety and adhere to professional codes of conduct [15]. While this is managed through informed consent, supervision, adherence to professional codes of conduct, and a constant focus on the trainee’s competence and ethical decision-making [15,16], it requires a high level of supervisory support, which can be resource-intensive for training programs managing increasing cohort sizes. The expansion of the psychological professions workforce places additional demands on supervision infrastructure, potentially affecting the quality and availability of supervisory support [1,14].

### Traditional Role-Play Methods in Mental Health Training

Role-play exercises are a cornerstone of competency-based training, offering structured opportunities to practice and receive feedback on specific therapeutic skills [17,18]. While role-plays provide valuable opportunities for skill development, they face substantial limitations in terms of ecological validity, scalability, and consistency [19,20]. Within psychological practitioner training, standardized role-plays using trained actors offer structured learning, though validity concerns persist [10]. Student-to-student role-plays face practical challenges such as inconsistencies in role-play quality due to student anxiety when role-playing with peers, authenticity limitations as students may deliberately facilitate their peers’ success rather than presenting realistic clinical challenges, ethical concerns regarding self-disclosure, and logistical difficulties across large student cohorts [17,18,21-23]. Research has identified 4 authenticity barriers, namely, students’ insufficient knowledge of psychiatric symptoms, reluctance to portray distressing emotions, artificial dynamics from preexisting relationships, and inadequate environmental settings [17,24]. Interestingly, studies show higher-performing students often experience greater anxiety during role-plays than lower-performing peers—a “competence-anxiety paradox” linked to skilled trainees’ greater awareness of therapeutic complexity [18]. Teacher demonstration models, where instructors model therapeutic techniques with volunteer “clients,” provide excellent teaching opportunities but limited hands-on practice for students [18]. These limitations contribute to the “theory-practice gap” where classroom knowledge fails to translate effectively to clinical application [20,25].

### Technological Innovations in Mental Health Training

Traditional role-play limitations have driven advancements in simulation technology for clinical skills training. Innovations included decision tree–based interactive videos that improved the standardization of training experiences and assessment procedures [26,27]. This approach has reduced psychological strain on actors playing patient roles, as they no longer needed to maintain complex character portrayals for extended periods and improved scalability by eliminating the need for extensive simulant training that required 10 hours for students or 3‐4 hours for clinicians [27]. While research shows skills can improve through these methods, there is variability in the degree to which students find interactive client systems engaging and useful [26,28]. Machine learning and language models have advanced patient simulations through contextual understanding and human-like text generation, which can increase realism comparable to human patients [29], across diverse scenarios, though responses sometimes appeared overly idealized [30]. While these approaches facilitate practice, they do not provide competency-based feedback to further skill development, which is essential for meeting professional training standards and ensuring therapists develop the specific competencies required for NHS service delivery.

### Rationale for This Study

Key gaps remain in the literature on virtual patient simulations for CBT training. First, to our knowledge, although existing tools have achieved realistic patient interactions [26,28-30], none have systematically translated established competence frameworks, such as the CTS-R or UCL competence frameworks, into real-time feedback during simulated practice. This limits their alignment with professional training standards and NHS service requirements. While artificial intelligence (AI) systems have been developed to assess CBT competence from recorded therapy sessions [31,32], the integration of such frameworks into simulation-based training represents a critical unmet need. Given that competence frameworks underpin evidence-based CBT training and professional accreditation [2,3], bridging this gap is essential for advancing scalable, standards-aligned training tools.

Approaches to operationalize competence development through frameworks and scales need to build on learning models such as experiential learning [33], deliberate practice methodologies [34], and technology-enhanced training [7,35]. Therefore, an AI-driven simulation platform that incorporates competency-based feedback mechanisms offers a promising solution to address the identified limitations in traditional training approaches while maintaining alignment with established professional frameworks and educational principles. Such a platform fosters experiential learning by enabling safe, active experimentation alongside structured reflection, while also supporting deliberate practice through systematic and repeatable skill development, representing a novel approach that addresses longstanding limitations in CBT training technology.

A second significant gap pertains to voluntary engagement with training technologies in psychological practitioner education. Existing studies on simulation-based learning have largely examined mandatory curricular components or controlled experimental settings, focusing primarily on perceived fidelity or effectiveness [28-30]. Understanding how trainees voluntarily engage with supplementary training technologies provides critical evidence for implementation feasibility and ecological validity. Unlike previous research, this study investigates naturalistic engagement patterns within real-world training programs, integration with existing curricula, and specific mechanisms through which such simulations might enhance learning. By combining quantitative engagement metrics with qualitative feedback, this research can provide preliminary evidence regarding the practical utility of AI simulations in psychological therapist training.

### Study Aims

This pilot study aimed to evaluate the educational potential of the first virtual patient platform providing competence framework–aligned feedback in psychological therapist training. Specifically, the study aimed:

1. to assess the acceptability of simulation-based learning integrated with competency-based feedback as a supplemental tool within established training programs for psychological therapists who use CBT in their practice.
2. to examine patterns of voluntary engagement with simulation-based practice among trainees from different professional training backgrounds.
3. to gather preliminary evidence regarding self-reported competence development following simulation-based practice experiences.
4. to explore trainees’ perceptions of the educational value of framework-aligned feedback during simulated therapeutic interactions compared to traditional training methods.

## Methods

### The CBT Trainer Platform

The CBT Trainer platform (TTZ) [36] is an AI-based virtual patient platform designed for mental health training. The app presented participants with interactive scenarios replicating therapeutic sessions for common mental health conditions, including depression, generalized anxiety disorder, social anxiety disorder, posttraumatic stress disorder, and agoraphobia. The platform primarily enabled voice- and text-based interaction with virtual patients exhibiting realistic clinical presentations. Users began by selecting from 6 virtual patients representing diverse demographics and clinical presentations. During voice- or text-based interactions, the system continuously evaluated responses against competence frameworks, providing real-time feedback visible in the interface.

**Figure 1.**
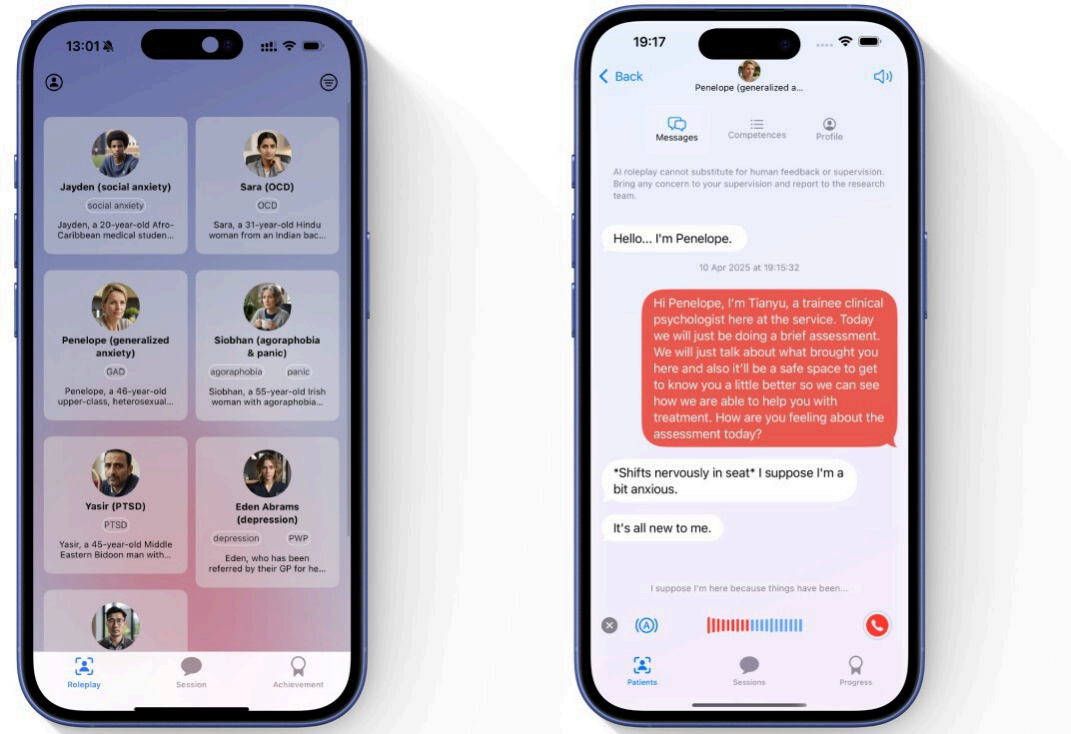
CBT Trainer patient selection (left) and role-play interfaces (right).

CBT Trainer represents the first virtual patient simulation to operationalize established competence frameworks into real-time automated feedback. The platform was designed to support the practice of both assessment and intervention skills. Users could select which type of session to practice (assessment or intervention) and choose the corresponding competence framework for feedback. The CBT Trainer assessed trainees against a selected competency scale—either the Cognitive Therapy Scale-Revised (CTS-R) [4] or the UCL Psychological Wellbeing Practitioner (PWP) Assessment Competence Scale [37]. CBT Trainer indicates whether specific competencies were demonstrated or required further development. After practice sessions, participants received structured feedback, highlighting competencies met and those not met, with specific guidance to support their development of key therapeutic competencies. The platform was designed to complement, not replace, traditional training methods by providing additional opportunities for independent skill practice. The CBT Trainer app’s content and features were kept unchanged throughout the data collection period to ensure consistency across all participant interactions. Figures1  2 illustrate the app’s user interface diagrams, including the patient selection screen displaying diverse clinical presentations, the interactive role-play screen, and the competency feedback screens.

**Figure 2.**
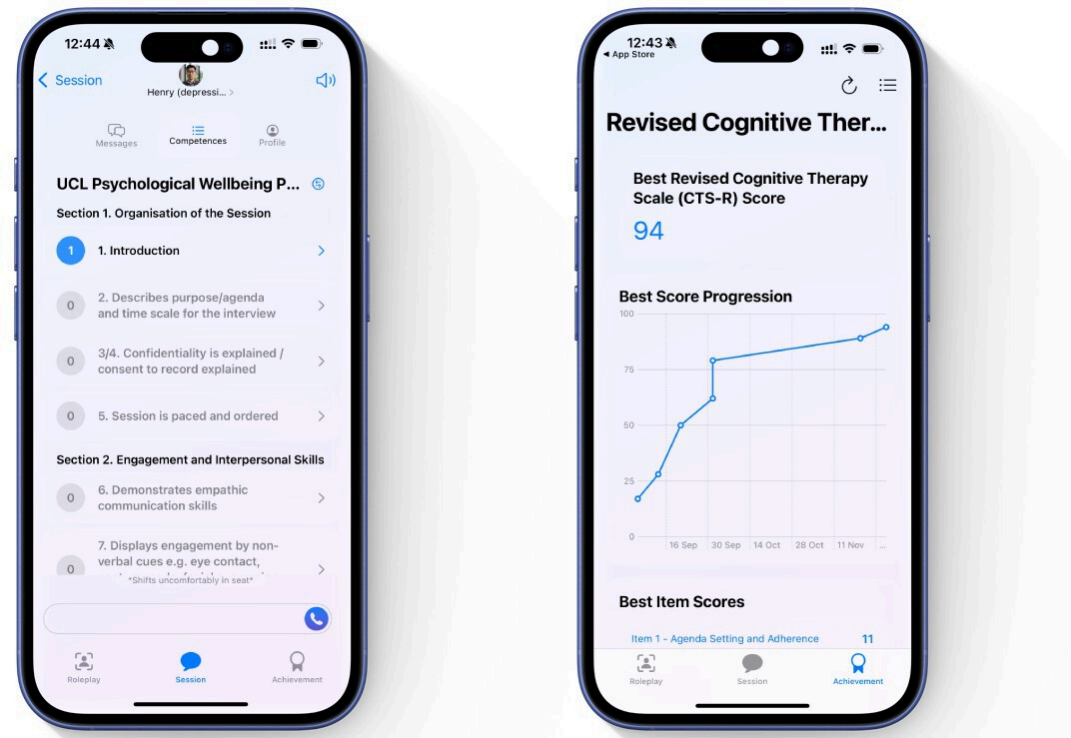
Competence assessment and progress tracking interfaces. (Left) University College London (UCL) Psychological Wellbeing Practitioner (PWP) Assessment Competence Scale interface shows real-time competence evaluation across core domains. (Right) Cognitive Therapy Scale-Revised (CTS-R) progress tracking screen.

### Platform Development and Validation

The CBT Trainer platform was developed in collaboration with UCL’s CORE, the research group that created the UCL competence frameworks upon which the platform is built. The development team comprised a clinical psychologist who designed patient profiles and competency-based feedback mechanisms, and a machine learning specialist who built the competence assessment algorithms.

Six diverse virtual patient profiles were created representing common mental health presentations, including depression, generalized anxiety disorder, social anxiety disorder, posttraumatic stress disorder, agoraphobia with panic, and obsessive-compulsive disorder (Table 1). Each profile was developed by a clinical psychologist to reflect realistic clinical presentations.

**Table 1.** Virtual patient profiles in CBT^a^ Trainer.

Name	Age (years)	Background	Primary presentation	PHQ-9^b^	GAD-7^c^
Henry	35	British Chinese, Liverpool	Depression with risk	21	18
Jayden	20	Afro-Caribbean, London	Social anxiety	12	16
Penelope	46	British, dyslexia	Generalized anxiety	15	19
Siobhan	55	Irish	Agoraphobia with panic	15	19
Yasir	56	Middle Eastern (Bidoon), asylum seeker	PTSD^d^	19	18
Sara	31	Indian Hindu, London	OCD^e^	17	19

a CBT: cognitive behavioral therapy.

b PHQ-9: Patient Health Questionnaire-9.

c GAD-7: Generalized Anxiety Disorder-7.

d PTSD: posttraumatic stress disorder.

e OCD: obsessive compulsive disorder.

Prior to the pilot study, the platform underwent iterative testing and refinement. Patient interactions were tested by course tutors from the Doctorate in Clinical Psychology and PWP training programs at UCL to assess clinical authenticity and educational appropriateness. Early testing revealed that initial response patterns provided too much information too quickly compared to authentic patient presentations (where patients disclose more over time as the therapeutic relationship evolves). Based on this feedback, we adjusted response length and detail level to match typical patient communication patterns.

Throughout development and deployment, several measures ensured appropriate platform responses. The system development includes alignment efforts focused on maintaining task adherence to therapeutic contexts. All feedback was explicitly grounded in established competence frameworks (CTS-R and UCL PWP Assessment Competence Scale), limiting assessment to observable, framework-specified competencies.

### Study Design

This pilot study used a 2-stage mixed methods approach to evaluate an AI-based virtual patient platform for training cognitive behavioral therapists. This included usability testing, digital engagement data, and questionnaires to assess acceptability and educational impact.

### Participants

Participants were recruited from psychological practitioner training programs at UCL during the 2024‐2025 academic year, targeting 2 specific programs, namely, the Doctorate in Clinical Psychology and the Low-intensity PWP training. Eligible participants were required to be enrolled in one of these programs and have access to an iOS device with internet connectivity capable of running the CBT Trainer app.

### Procedure

The study was conducted in 2 stages. Stage 1 involved 4 participants in 1-hour in-person usability testing sessions and engaged with the platform under the researcher’s observation. This allowed us to examine CBT trainer functionality and intuitiveness based on user actions and responses. Stage 1 findings informed Stage 2 by identifying needed platform refinements (eg, user experience improvements and bug fixes).

Stage 2 expanded the study to include 101 participants who completed an initial online screening. After completing online screening, informed consent, and demographic questionnaires, eligible participants downloaded the app to their personal device. Participants were encouraged to use the platform for at least 60 minutes during the study period to gain meaningful experience, though they were explicitly informed that there was no mandatory usage requirement and they could engage with the app as much or as little as they preferred. Two reminder emails were sent to all participants during weeks 1 and 3 of the 4-week study period, providing tips on integrating the platform with their training program activities. The stage concluded with a comprehensive online survey.

All 101 participants completing the screening survey were entered into a draw for 1 of 3 £45 (approximately US $59) vouchers, but no incentives were provided for app download or engagement to preserve the ecological validity of usage patterns.

The research version of the CBT Trainer app was distributed through Apple’s TestFlight beta testing platform to maintain separation between research data and any future public release, which required participants to complete multiple steps, including receiving and accepting email invitations, downloading the TestFlight app, and then installing CBT Trainer.

### Measures

#### Survey Instruments

##### System Usability Scale

A standardized 10-item questionnaire measuring platform usability, with scores ranging from 0 to 100 [38]. Scores above 68 are considered above average, and scores above 80 are considered excellent [39].

##### PostStudy Questionnaire

A custom questionnaire designed to capture participants’ experiences with the CBT Trainer platform. The questionnaire comprised four main sections: (1) 3 subjective learning outcome measures assessing helpfulness, skill improvement, and perceived realism using 0‐100 rating scales; (2) a checklist assessment of specific competence development across therapeutic domains; (3) comparative evaluation questions rating CBT Trainer against traditional role-play methods; and (4) open-ended qualitative questions exploring learning impact and user experience. The complete poststudy questionnaire is available in Multimedia Appendix 1.

### Engagement Metrics

Platform usage was tracked through several dimensions, including role-play time (time spent in active interaction with simulated patients), session quantity (total number of role-play sessions created per user), and interaction depth (average exchanges between trainees and virtual patients per session, where each exchange comprised 1 trainee input paired with 1 AI patient response).

### Data Analysis

Descriptive statistics were computed for all continuous variables (eg, System Usability Scale [SUS] scores, engagement metrics, and self-rated learning outcomes) and are presented as means, SDs, medians, and ranges. Categorical data (eg, demographic variables, competence development checklist items, and comparative advantages) are presented as frequencies (n) and percentages (%). Missing data were addressed by using complete case analysis, resulting in varying sample sizes across different analyses, which are explicitly reported alongside each result (eg, n=33 for SUS scores and n=31 for competence ratings).

Qualitative responses from an open-ended survey question, “Please provide one or two specific examples of how CBT Trainer impacted your clinical skills or knowledge,” were analyzed using qualitative content analysis [40,41]. Qualitative content analysis was chosen as it enables describing and quantifying qualitative data while preserving participants’ expressed experiences—appropriate for this acceptability study where we sought to identify the frequency and range of participant perspectives on platform utility while preserving participants’ expressed experiences [42]. Analysis focused on identifying manifest (ie, surface level) meanings in the data following an inductive approach [43]. Analysis was conducted by TTZ, a clinical psychology doctoral research student and one of the platform developers, who maintained reflexive awareness of potential bias throughout the analytic process. The questionnaire responses were read several times by TTZ. All descriptions of specific ways the CBT Trainer impacted participants’ clinical skills and training experiences were considered meaning units. If an impact was described multiple times within a single participant’s response, these descriptions were conjoined into a single meaning unit. Codes (ie, specific impacts on clinical skills and knowledge) were then grouped into categories and subcategories on the basis of similarities and differences in their educational and learning functions.

### Outcomes

The research team established prespecified outcomes prior to study commencement, following implementation of study guidelines [44]:

- Primary outcomes

- Platform engagement: target of >50% of participants using the app for ≥10 minutes, or >25% using it for ≥30 minutes (Aim 2)
- Perceived educational value: target of >70% of participants reporting positive impact on training (Aim 4)

- Secondary outcomes

- Recruitment and retention: target of ≥22 participants across both study phases (Aim 1)
- System usability: target SUS score >68 (Aim 1)

- Exploratory

- Self-reported improvements across competency domains (Aim 3)

### Ethical Considerations

The study received approval from the UCL Research Ethics Committee (21883/006). The study protocol was preregistered with the Open Science Framework [45].

Several safeguards were implemented to address ethical concerns. Course directors from participating training programs were aware of the study. Meetings with program staff occurred at study commencement and debriefing. A human-in-the-loop design was implemented with weekly research team meetings reviewing engagement patterns to identify any concerning patterns. Specifically, the team monitored for (1) misuse of the platform for noneducational purposes through sampled transcript review, (2) inappropriate content or off-task interactions, (3) technical issues (error reports and crashes) that could impede learning, and (4) instances where AI feedback appeared inappropriate or misaligned with competence frameworks. No significant concerning engagement patterns requiring intervention were identified during the study period. Reminder emails sent at weeks 1 and 3 provided opportunities to report concerns. The platform included a direct link to the study information sheet. Participants were informed that the platform was experimental and supplementary to the traditional training, with in-app reminders to bring concerns to their supervisors and reminders of the limitations of AI-generated competency feedback. Survey questions provided opportunities for participants to report any concerns or difficulties. Support was available from researchers for technical issues or concerns about platform feedback. Participants who ceased engagement were contacted during survey collection to check whether issues had arisen. Coercion was mitigated through voluntary self-selection recruitment via email advertisement, no incentive payments for engagement, explicit consent procedures reminding that no minimum usage is required, and reminding the right to withdraw. Data privacy and security were prioritized, with personal identifiers kept separate from research data. Multiple contact pathways were provided within the app, including principal investigator contact, ethics committee contact, and UCL Data Protection Office contact.

## Results

### Phase 1: Initial Usability Testing

Phase 1 involved 4 doctoral clinical psychology students (all women, aged 25‐34 years). PWP trainees were not included in Phase 1 usability testing due to their later program start date. Participants engaged with the platform under the researcher’s observation. Researchers monitored interactions for technical issues and clinical appropriateness. Several bugs and user experience navigation issues were revealed and fixed before Phase 2 implementation.

### Phase 2: Extended Implementation

#### Participant Characteristics

**Figure 3.**
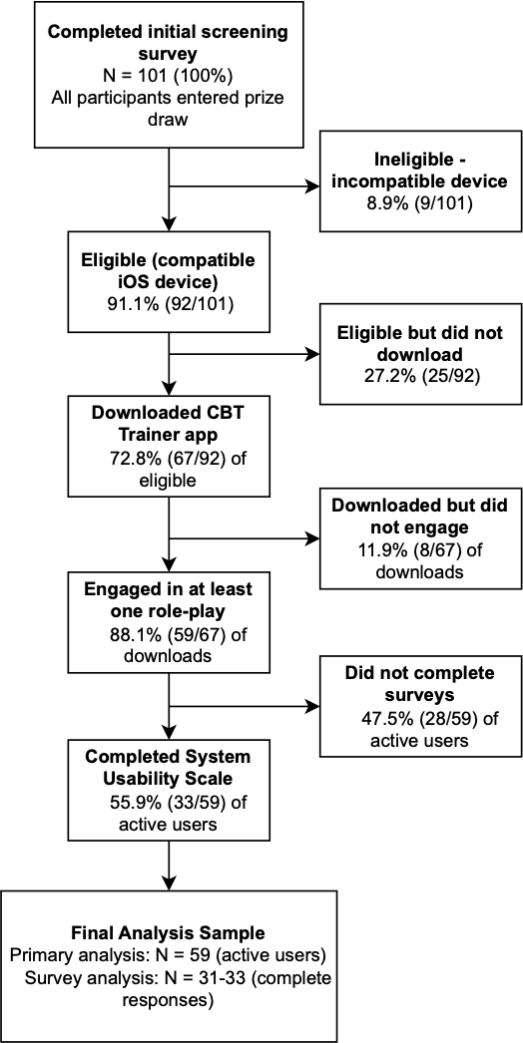
Participant flow diagram; CBT: cognitive behavioral therapy.

Figure 3 illustrates participant flow and attrition throughout the study phases. Of 101 invited, 92 (91.1%) were eligible based on iOS device compatibility. Attrition occurred primarily at app download, where 25 eligible participants (27.2%) did not proceed likely due to TestFlight app download complexity and technical barriers, and at survey completion, where 28 active users (47.5%) did not complete follow-up assessments, likely mistaking the end-of-study survey as optional educational monitoring rather than mandatory research data collection. Technical difficulties with older iOS devices prevented 8 participants (8.7% of the eligible) from engaging despite successful installation. Overall, 59 participants (64.1% of the eligible) successfully engaged with the platform, with 31‐33 completing comprehensive survey assessments for secondary analyses.

Of the 59 engaging participants, 84.7% (n=50) are from the Low Intensity Cognitive Behavioral Interventions program and 15.3% (n=9) are from the Doctorate in Clinical Psychology program. Participants were predominantly women (49/59, 83.1%) with a mean age of 28.37 (SD=7.25; range 22‐62) years. The ethnic distribution included White (34/59, 57.6%), Black or African or Caribbean or Black British (9/59, 15.3%), Asian or Asian British (7/59, 11.9%), Other ethnic groups (5/59, 8.5%), and Mixed or Multiple ethnic groups (4/59, 6.8%). The majority of participants were at the early stages of their training, with a mean enrollment time of 1.61 months in their respective programs (SD 2.65; median 1.0, IQR 0‐18 months). Regarding familiarity with competency assessment frameworks, 39% (n=23) reported being somewhat familiar, 25.4% (n=15) were neutral, 16.9% (n=10) were somewhat unfamiliar, 16.9% (n=10) were very unfamiliar or had never heard of them, and 1.7% (n=1) were very familiar. Exploratory analyses revealed no substantial differences in engagement patterns, demographics, or outcomes between the 2 training programs, supporting combined analysis of the sample.

#### Platform Engagement

Of the 59 engaging participants, they spent an average of 95.24 (SD 134.58; median 45.34, IQR 11.57-105.15) minutes engaging in role-plays. Users created an average of 4.24 role-play sessions each (SD 3.72; range 1‐14), with an average session duration of 21.48 (SD 23.24; median 14.21, IQR 5.93-32.82) minutes. Each session contained an average of 48.58 (SD 57.11, median 21, IQR 4–82) interactions. The data were right-skewed (most values clustered at the low end with a long tail of high values). While most participants engaged for shorter periods, a smaller number engaged for very long periods (several hours).

### System Usability

The CBT Trainer platform achieved high usability ratings with a mean SUS score of 82.2 (SD 12.93; median 82.5, IQR 72.5-92.5, n=33), placing it in the “excellent” category according to established benchmarks [39].

### Self-Reported Competence Development

**Figure 4.**
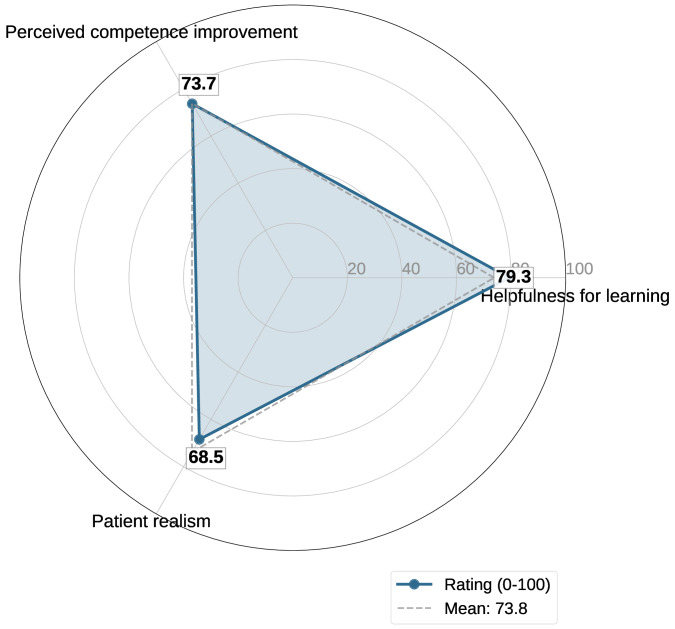
Educational impact ratings reported by CBT (cognitive behavioral therapy) Trainer users (n=31).

Participants reported high agreement that “The simulated patient interactions were helpful for my learning” (mean 79.35, SD 17.49; median 80.0, IQR 69.5-95.5, n=31) and “My clinical skills has improved after using CBT Trainer” (mean 73.67, SD 23.03; median 81.0, IQR 60.0-90.0 ,n=30), as illustrated in Figure 4. The statement “The simulated patients were realistic to real patients” received moderately strong agreement (mean 68.45, SD 20.45; median=70.0, IQR 49.5-87.5, n=31).

**Figure 5.**
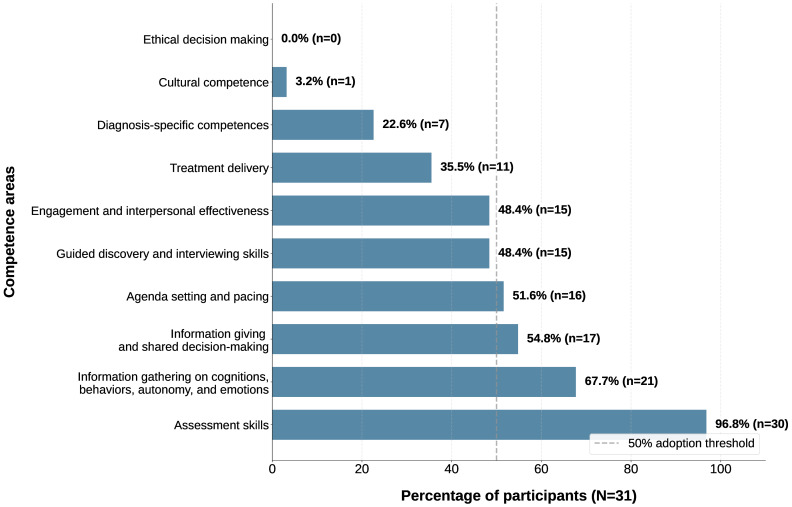
Competence development areas reported by CBT (cognitive behavioral therapy) Trainer users.

The distribution of self-reported competence development varied considerably across the assessed domains (Figure 5). Some competency areas showed limited improvement, including ethical decision making (0/31, 0%) and cultural competence (1/31, 3.2%). The most frequently reported competence improvements (Figure 5) were in assessment skills (30/31, 96.8%), followed by information gathering on cognitions, behaviors, autonomy, and emotions (21/31, 67.7%), and information giving and shared decision-making (517/31, 4.8%).

### Qualitative Feedback on Learning Impact

Content analysis of qualitative feedback gathered through the open-ended question “Please provide one or two specific examples of how CBT Trainer impacted your clinical skills or knowledge” (n=21 responses) in the poststudy questionnaire revealed 38 distinct codes describing specific impacts on clinical skills and knowledge. These codes were clustered into 8 main categories, with a full description of each code provided in Multimedia Appendix 2.

#### Category 1: Skill Development

Participants reported improvements in specific clinical techniques, communication skills, assessment abilities, and session management. This was the most frequently cited category (16/21, 76.2%). Reported improvements in specific clinical techniques include risk assessment (4/21, 19%), funneling (3/21, 14.3%), and structured information gathering using frameworks like 4Ws and ABCs (2/21, 9.5%). As one participant described:

*The function of being able to analyse your conversation with the patient and compare against the specific competencies was incredibly helpful... This really helped me to improve my information gathering skills using the 4Ws and ABCs.”*

[P19]

Communication skills improvements included developing more flexible and adaptive responses (2/21, 9.5%) and refining questioning styles (2/21, 9.5%). One participant noted:

*Normally I stutter when talking to patients and this helped me to think before I speak*

[P12]

Many participants reported enhanced assessment skills, both generally (4/21, 19%) and specifically in understanding competency-based assessment (4/21, 19%). Improvements in session management were frequently described, including better pacing (4/21, 19%), time management (2/21, 9.5%), and session structure (2/21, 9.5%). Participant explained:

*It also really helped me to improve my pacing of the assessment, as I was able to look at my timings afterwards to work out how much time I spent in each section.*

[P19]

#### Category 2: Practice Accessibility and Autonomy

Nearly half of the participants (9/21, 42.9%) valued the accessibility and autonomy the platform provided. The most commonly cited benefit was independent practice (7/21, 33.3%), which reduced dependence on peers or family members. As one participant described:

*I found it difficult to continuously practise with my friends and family, and it was a bit unfair, as it would take about 45mins of their free time... Being able to use CBT Trainer meant that I could practise an entire assessment repeatedly.*

[P3]

Flexible scheduling (4/21, 19%) and the ability to engage in repeated practice (3/21, 14.3%) were also valued features.

#### Category 3: Feedback and Competence Assessment

More than two-thirds of participants (13/21, 61.9%) referenced the value of structured feedback aligned with competence frameworks. Competence gap identification was the most frequently mentioned benefit (8/21, 38.1%), followed by competence framework alignment (6/21, 28.6%). A participant explained:

*It gave me an understanding of where I was and wasn’t hitting the mark scheme. For example, where there wasn’t enough evidence of covering the confidentiality criteria.*

[P10]

Constructive, personalized feedback was valued by several participants (5/21, 23.8%), with Participant 19 noting the platform “gave personalized tips for each competence.”

#### Category 4: Confidence, Preparedness, and Anxiety Reduction

More than half of participants (11/21, 52.4%) reported that the CBT Trainer enhanced their confidence and preparedness while reducing anxiety. Exam and OSCE preparation (5/21, 23.8%) and confidence building (5/21, 23.8%) were equally prominent. Participant described:

*Knowing that I could practice anytime of the day made me feel so much more calm about my exam as I didn’t have to rely on other people to practice with.*

[P15]

Anxiety reduction was specifically mentioned by 3 participants (14.3%), while preparation for clinical placements was noted by 2 (9.5%).

#### Category 5: Psychological Safety and Judgment-Free Learning

A small number of participants (2/21, 9.5%) explicitly referenced the psychological safety the platform provided. Participant 1 valued being able to “practice and experiment with new techniques and skills during training without fear of judgment.”

#### Category 6: Diversity and Presentation-Specific Learning

Approximately one-third of participants (6/21, 28.6%) valued exposure to diverse patient presentations (4/21, 19%) and different presenting problems (3/21, 14.3%). Participant 15 noted: “The diversity of the patients was also helpful to prepare for real life.” Some participants specifically valued practice with challenging scenarios (3/21, 14.3%) and opportunities for culturally sensitive practice (1/21, 4.8%), such as working with patients from different religious backgrounds.

#### Category 7: Reflection and Self-Awareness

Over one-third of participants (7/21, 33.3%) reported enhanced reflection and self-awareness. Self-awareness of performance was the most common impact (5/21, 23.8%), followed by the ability to engage in conversation analysis (3/21, 14.3%). One participant described:

*The competence section was extremely helpful as it helped me to recognize areas I missed or did not go over during my roleplay.*

[P20]

#### Category 8: No Perceived Impact

One participant (4.8%) reported no perceived impact on clinical skills or knowledge from using CBT Trainer. Participants also identified limitations of the platform. The most frequently cited limitation was the absence of nonverbal communication cues (7/21, 33%), with participants noting they “can’t read body language” (P15) and missing “micro expressions” that inform real clinical work. Several participants found patient responses occasionally unrealistic (5/21, 24%), describing virtual patients as “too willing to engage compared to real clients” (P9) or “overly positive about interventions” (P1). Technical issues with voice recognition were mentioned by 14% (n=3), and some desired greater diversity in clinical presentations (2/21, 10%).

### Comparison to Traditional Role-Play

**Figure 6.**
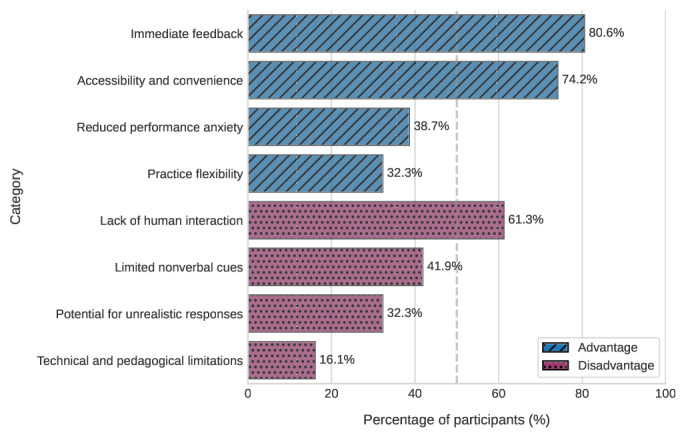
Perceived advantages and disadvantages of CBT Trainer compared to traditional training methods by CBT (cognitive behavioral therapy) Trainer users (n=31).

Participants were asked to evaluate “How does CBT Trainer compare to traditional role-play exercises with peers? (0=Traditional methods much better, 10=CBT Trainer much better)” and rated CBT Trainer against traditional classroom role-play with a mean score of 5.90 (SD 1.94; median 6.0, IQR 5.0-6.0, n=31) on a 10-point scale favoring CBT Trainer, indicating moderate comparability. Figure 6 shows participants’ perceptions of the comparative advantages and disadvantages of CBT Trainer relative to traditional role-play methods.

### Primary and Secondary Outcomes

All prespecified implementation targets were achieved. For platform engagement, 79.66% (n=47) of 59 active users engaged with the platform for ≥10 minutes, and 59.32% (n=35) used it for ≥30 minutes, both exceeding target thresholds. Perceived educational value similarly exceeded the target of >70% reporting positive impact (rating >50/100) across all 3 educational value statements: 90.3% (28/31) for simulated patient interactions being helpful for learning, 71.0% (22/31) for simulated patients being realistic to real patients, and 83.3% (25/30) for clinical skills improved after using CBT Trainer of participants rating each statement above 50/100, respectively. Recruitment and retention substantially exceeded targets with 101 initial participants and 59 active users. The mean SUS score of 82.2 (SD 12.93) was well above the target threshold of >68.

## Discussion

### Principal Findings

This pilot study evaluated CBT Trainer, the first AI-driven virtual patient platform to provide real-time feedback aligned with established competence frameworks (CTS-R and UCL PWP Assessment Scale) in psychological practitioner training. It is also the first study to examine voluntary engagement patterns with such simulations within real-world training programs. The platform demonstrated strong feasibility and acceptability, with most participants voluntarily engaging in extended practice sessions—indicating meaningful opportunities for therapeutic skill development. Excellent usability ratings were achieved, and participants reported substantial educational value, particularly in foundational clinical skills. Self-reported improvements were most notable in assessment competencies, where nearly all participants reported enhanced assessment skills and most noted gains in information gathering. Qualitative analysis revealed that participants valued the platform’s immediate feedback, convenience, and judgment-free practice environment as key advantages over traditional peer role-plays, while acknowledging limitations such as the absence of nonverbal cues. We discuss these results in relation to existing literature, consider their implications for psychological training, and discuss limitations and future research directions.

### Feasibility and Acceptability (Aim 1)

The study provides evidence supporting the acceptability and feasibility of implementing AI-based virtual patient simulations with competency-aligned feedback in psychological therapist training programs that use CBT. Participant engagement exceeded prespecified targets, with substantial uptake and active involvement in role-play interactions. The platform achieved excellent usability ratings, further supporting its feasibility for integration into clinical training environments [39]. These findings align with prior research on AI-based simulated patients in health care education [26,29] and extend them to psychological therapist trainees who use CBT specifically, demonstrating that this population similarly values interactive simulations as learning tools.

### Patterns of Voluntary Engagement (Aim 2)

During the voluntary 1-month access period, engagement patterns were diverse, reflecting individualized approaches to simulation-based learning. While most participants opted for shorter, focused practice sessions, a smaller subset engaged in extended periods of use, spanning several hours. This variability suggests that trainees may adopt distinct engagement strategies—whether for brief skill rehearsal or more immersive, in-depth practice. The duration and interaction density of role-play sessions were substantial enough to facilitate meaningful skill development across multiple competency domains. No substantial differences were observed in engagement patterns, demographics, or outcomes between the 2 training programs. This overall engagement profile indicates that trainees perceived the platform as a valuable supplement to traditional training and that the simulations provided a sufficiently authentic approximation of therapeutic dialogue in both depth and communicative substance.

### Self-Reported Competence Development (Aim 3)

Participants reported substantial improvement in assessment skills and information gathering, indicating that the platform effectively supports the development of fundamental therapeutic competencies. These improvements in core clinical skills, supported by strong ratings for educational value and perceived clinical skill improvement, mirror a similar study that observed significant gains in reflection skills among users of their patient-like conversational agent [28]. The reported benefits extended beyond assessment to include domains such as risk assessment, therapeutic pacing, and interpersonal effectiveness, suggesting that virtual patient interactions can facilitate development across multiple competence areas, in line with evidence that structured AI simulations can enhance targeted clinical skills [46]. However, the variability in perceived improvement competency domains implies that certain therapeutic skills may be more amenable to technological simulation than others, pointing to both the potential and the boundaries of AI-driven training tools.

### Educational Impact and Perceived Value (Aim 4)

#### Self-Reported Educational Impact

CBT Trainer’s capacity to facilitate immediate reflection on therapeutic interactions aligns with the principles of experiential learning [33], particularly enhancing the critical reflective observation and abstract conceptualization phases that bridge concrete experience and active experimentation. This immediacy addresses a fundamental requirement of deliberate practice [34], where skill acquisition depends on timely opportunities for reflection and refinement following performance attempts.

Participants consistently highlighted the benefit of practicing in a judgment-free environment. This aspect addresses several persistent challenges in traditional training, including performance anxiety during role-plays [18,22], evaluation apprehension inhibiting reflection [47] and delayed feedback cycles that hamper systematic improvement of therapeutic microskills [7,48]. Participants’ emphasis on the judgment-free practice environment suggests that the platform can mitigate these barriers concurrently, thereby fostering the deliberate, reflective skill development that is often constrained in conventional training formats.

#### Comparative Advantages and Challenges

The balanced perspective offered by participants when comparing AI-based practice to traditional role-play methods highlights the complementary nature of these approaches. When asked about the platform’s strengths, participants most frequently identified immediate feedback and convenience as significant educational benefits. Many users also valued the reduced performance anxiety that virtual practice offered compared to peer-based role-plays. Consistent with CBT principles of graded exposure, the platform may serve as an initial step that builds foundational skills before trainees progress to potentially more anxiety-provoking live role-play practice. The pattern of responses regarding comparative advantages aligns with the “pedagogical affordances” framework [49], which suggests that different educational technologies offer unique benefits for specific learning objectives. The participants’ nuanced assessment of when and how the platform added value to their training reflects a sophisticated understanding of how technological tools fit within their broader educational experience.

Participants also recognized certain limitations of the technology, particularly regarding interpersonal elements. The recognized lack of nonverbal cues and potential for unrealistic responses echo concerns raised regarding the authenticity of AI-based clinical simulations [30]. These limitations reinforce that current AI technology serves as a complement to, rather than a replacement for, conventional training models incorporating interpersonal engagement and supervision.

### Limitations and Future Directions

Our findings should be interpreted with several limitations in mind. Technical limitations restricting participation to iOS users may have introduced selection bias. A substantial proportion of eligible participants who initially expressed interest did not progress to downloading or using the app. This pattern of attrition suggests the importance of understanding barriers to technology adoption in clinical training contexts, whether technical, motivational, or practical in nature. Future research should systematically investigate factors influencing technology adoption among clinical trainees, including surveys of nonusers to identify specific barriers and preferences.

Participants were predominantly early-stage trainees who had not yet received formal training in intervention techniques. This may explain why self-reported improvements were concentrated in assessment and information gathering skills rather than intervention delivery competencies. In addition, participants were trainee PWPs and clinical psychologists using CBT, not trainees from CBT-specific programs. Future research should evaluate the platform’s utility in specialized CBT training contexts.

Methodologically, our mixed methods approach provided rich experiential data, but the absence of a control group prevents causal inferences about the platform’s impact on skill development. The study relied on self-reported skill improvement rather than objective competence assessment, weakening conclusions about educational efficacy. The 1-month evaluation period offers limited insight into long-term engagement patterns. While exploratory analyses revealed no substantial differences in engagement between the 2 training programs, we did not systematically examine how prior CBT experience might influence engagement patterns. It is plausible that trainees with more CBT experience might engage differently than those new to CBT, either showing reduced engagement due to perceived redundancy or increased engagement due to greater appreciation of competence frameworks. Future evaluations should incorporate standardized competence evaluations, longitudinal designs across training stages, control of prior experience, and randomized controlled trials.

While the platform operationalized general CBT competencies through established frameworks such as the CTS-R and UCL PWP Assessment Scale, it did not explicitly integrate disorder-specific evidence-based models into its feedback mechanisms. For example, in interactions with the virtual patient presenting with generalized anxiety disorder, the platform assessed competencies such as application of change methods (CTS-R item 11) but did not evaluate whether the trainee’s intervention strategies aligned with a specific evidence-based model for generalized anxiety disorder (eg, the Dugas intolerance of uncertainty model). Future studies could incorporate disorder-specific competence criteria and enable feedback on the appropriateness and fidelity of evidence-based intervention models, thereby enhancing their alignment with specialized CBT training requirements.

While we implemented several safeguards, the current model still places some responsibility on trainees to identify inappropriate feedback. This represents a limitation, as novice trainees may lack the expertise to critically evaluate AI-generated feedback. Future implementations should consider automated feedback auditing systems, routine expert review of AI-generated feedback, and structured guidance for trainees on how to critically engage with AI feedback within supervision.

While AI-driven simulations offer significant benefits for CBT training, their deployment must address known risks, including algorithmic bias, privacy concerns, and overreliance on technology that could erode professional judgment [50]. Comprehensive evaluation of AI training tools requires continued attention to fairness metrics, cultural appropriateness across diverse populations, and environmental sustainability—areas warranting further research as the field evolves.

The core methodology—AI simulation with competence framework–aligned feedback—may extend beyond CBT to other health care disciplines with established competence frameworks, including medicine, nursing, social work, and allied health professions. Adaptation would require profession-specific scenarios, alignment with relevant competence standards, and validation that simulation skills transfer to clinical practice. Disciplines with well-defined assessment criteria would be particularly suited to this approach, and empirical evaluation across training contexts is needed.

### Conclusions

The pilot study demonstrates that CBT Trainer—an AI-driven virtual patient platform delivering real-time competency-aligned feedback—is both acceptable and feasible within psychological training programs that use CBT. Participants engaged voluntarily and extensively with the platform, reporting high usability and significant educational value, particularly in core assessment and information gathering skills. Qualitative analysis revealed that participants highly valued the competence framework–aligned feedback, a judgment-free practice environment that reduced anxiety, and the flexibility for independent, repeated skill rehearsal. These features were seen as key advantages over traditional peer-based role-plays. The findings indicate that AI-based virtual patient simulations with competence framework–aligned feedback can effectively complement traditional training methods, particularly for developing foundational clinical skills in a flexible, accessible format. Supplementary tools like this can be valuable in contexts facing supervision shortages or growing demand for mental health services. We recommend phased adoption with appropriate oversight mechanisms while simultaneously conducting the rigorous trials necessary to build the evidence base. Additionally, we emphasize the importance of involving course leaders, clinical supervisors, competency scale and framework experts, and policymakers in the ongoing refinement and validation. Future research should use randomized controlled designs with objective competence assessments and evaluate the platform’s utility in specialized mental health training programs with more advanced trainees.

## Supplementary material

10.2196/84091 Multimedia Appendix 1 Poststudy survey instruments.

10.2196/84091 Multimedia Appendix 2 Description and frequencies of impact codes.

## Abbreviations

AI: artificial intelligence
CBT: cognitive behavioral therapy
CORE: Center for Outcomes Research and Effectiveness
CTRS: Cognitive Therapy Rating Scale
CTS-R: Cognitive Therapy Scale-Revised
NHS: National Health Service
PWP: Psychological Wellbeing Practitioner
SUS: System Usability Scale
UCL: University College London
