# Supplementary material for: An AI-Driven Virtual Patient Platform (CBT Trainer) for Training Cognitive Behavioral Therapy Practitioners Against Competencies: Mixed Methods Pilot Study
Source: JMIR Med Educ. 2026 Mar 6;12:e84091. doi: 10.2196/84091 (PMC12978919; doi:10.2196/84091)
Supplement: Multimedia Appendix 1 [file mededu-v12-e84091-s001.docx]

# Multimedia Appendix 1: Post-Study Survey Instruments

## Section 1: Usability Assessment

### System Usability Scale (SUS)

Participants rated the following 10 items on a 5-point Likert scale (1 = Strongly disagree, 5 = Strongly agree):

1. I think that I would like to use Patient AI frequently.
2. I found the system unnecessarily complex.
3. I thought the system was easy to use.
4. I think that I would need the support of a technical person to be able to use this system.
5. I found the various functions in this system were well integrated.
6. I thought there was too much inconsistency in this system.
7. I would imagine that most people would learn to use this system very quickly.
8. I found the system very cumbersome to use.
9. I felt very confident using the system.
10. I needed to learn a lot of things before I could get going with this system.

## Section 2: Competence Development

### Competence Checklist (Multiple Selection)

Which specific competencies do you feel the app helped you develop?

- Assessment skills
- Treatment delivery
- Agenda setting and pacing
- Engagement and interpersonal effectiveness
- Information gathering on cognitions, behaviours, autonomy and emotions
- Guided discovery and interviewing skills such as Socratic questioning and funnelling
- Information giving and shared decision making
- Diagnosis-specific competences
- Cultural competence
- Ethical decision making
- Other (please specify)

## Section 3: Self-Reported Learning Outcomes

### Impact Rating Scale (0-100 slider scale)

Participants rated agreement with the following statements on a 0-100 scale (0 = Fully disagree, 100 = Fully agree):

1. The simulated patient interactions were helpful for my learning.
2. The simulated patients were realistic to real patients.
3. My clinical skills have improved after using Patient AI.

## Qualitative Question on Clinical Impact

Please provide one or two specific examples of how Patient AI impacted your clinical skills or knowledge. *(Open-ended text response - source for qualitative content analysis)*

## Section 4: Comparison to Traditional Training Methods

### Comparative Rating

On a scale of 0-10, how does Patient AI compare to traditional role-play exercises with peers? *(0 = Traditional methods much better, 10 = Patient AI much better)*

### Comparative Advantages (Select up to 3)

What are the most prominent advantages that Patient AI offers you over traditional training methods?

- Convenience
- Variety of cases
- Immediate feedback
- Reduced performance anxiety
- Ability to repeat scenarios
- Standardized experiences
- Self-paced learning
- Other (please specify)

### Comparative Disadvantages (Select up to 3)

What are the most prominent disadvantages for your using Patient AI compared to traditional training methods?

- Lack of human interaction
- Limited non-verbal cues
- Potential for unrealistic responses
- Technical limitations (iPhone/iPad)
- Less adaptable to individual needs
- Reduced supervisor involvement
- Overreliance on technology
- Other (please specify)
