# Supplementary material for: An AI-Driven Virtual Patient Platform (CBT Trainer) for Training Cognitive Behavioral Therapy Practitioners Against Competencies: Mixed Methods Pilot Study
Source: JMIR Med Educ. 2026 Mar 6;12:e84091. doi: 10.2196/84091 (PMC12978919; doi:10.2196/84091)
Supplement: Multimedia Appendix 2 [file mededu-v12-e84091-s002.docx]

# Multimedia Appendix 2

| **Category/Code** | **Description** | **Frequency, N (%)** |
| --- | --- | --- |
| **SKILL DEVELOPMENT & TECHNIQUE MASTERY** |  | **16 (76.2%)** |
| *Specific Clinical Techniques* |  |  |
| Funneling | Improved ability to funnel questions and gather focused information | 3 (14.3%) |
| Information gathering (4Ws and ABCs) | Enhanced use of structured information gathering frameworks | 2 (9.5%) |
| Risk assessment | Improved skills in conducting risk assessments conversationally | 4 (19.0%) |
| Safety planning | Enhanced ability to develop safety plans with clients | 1 (4.8%) |
| *Communication Skills* |  |  |
| General communication | Overall improvement in therapeutic communication | 1 (4.8%) |
| Flexible/adaptive responses | Increased ability to adjust responses based on patient input | 2 (9.5%) |
| Speech fluency | Improved fluency and reduced stuttering when speaking | 1 (4.8%) |
| Questioning style | Development of effective questioning techniques | 2 (9.5%) |
| *Assessment Skills* |  |  |
| General assessment skills | Overall improvement in clinical assessment abilities | 4 (19.0%) |
| Competence-based assessment | Understanding of competence criteria and frameworks | 4 (19.0%) |
| *Session Management* |  |  |
| Session pacing | Improved ability to pace sessions appropriately | 4 (19.0%) |
| Time management | Better awareness and management of session timing | 2 (9.5%) |
| Session structure | Enhanced organization of session components | 2 (9.5%) |
| CBT-specific techniques | Improved discussion of cognitions and behaviours | 1 (4.8%) |
| **PRACTICE ACCESSIBILITY & AUTONOMY** |  | **9 (42.9%)** |
| Independent practice | Ability to practice without relying on peers or family | 7 (33.3%) |
| Flexible scheduling | Practice at own pace and convenient times | 4 (19.0%) |
| Repeated practice | Opportunity for multiple practice sessions | 3 (14.3%) |
| Alternative to peer practice | Substitute for difficult-to-arrange peer role-plays | 3 (14.3%) |
| Breaking down sections | Ability to focus on specific session components | 1 (4.8%) |
| **FEEDBACK & COMPETENCE ASSESSMENT** |  | **13 (61.9%)** |
| Competence framework alignment | Feedback aligned with formal competence scales | 6 (28.6%) |
| Competence gap identification | Recognition of specific areas needing improvement | 8 (38.1%) |
| Specific criterion-referenced feedback | Detailed feedback on specific competence criteria | 3 (14.3%) |
| Constructive/personalized feedback | Tailored suggestions for improvement | 5 (23.8%) |
| Self-assessment capability | Ability to evaluate own performance against criteria | 4 (19.0%) |
| Immediate feedback | Receiving feedback during or immediately after practice | 2 (9.5%) |
| Identifies areas to explore | Highlights aspects requiring deeper investigation | 1 (4.8%) |
| **CONFIDENCE, PREPAREDNESS & ANXIETY REDUCTION** |  | **11 (52.4%)** |
| Exam/OSCE preparation | Better prepared for assessments and examinations | 5 (23.8%) |
| Confidence building | Increased self-assurance in clinical skills | 5 (23.8%) |
| Anxiety reduction | Reduced nervousness about exams and clinical practice | 3 (14.3%) |
| Placement preparation | Better prepared for clinical placements | 2 (9.5%) |
| Reduced reliance on others | Less dependent on peers for practice and preparation | 2 (9.5%) |
| Script development | Developing comfortable conversational approaches | 1 (4.8%) |
| **PSYCHOLOGICAL SAFETY & JUDGMENT-FREE LEARNING** |  | **2 (9.5%)** |
| Practice without fear of judgment | Safe environment free from peer evaluation | 2 (9.5%) |
| Experimentation with techniques | Freedom to try new approaches without consequences | 1 (4.8%) |
| Private/controlled environment | Safe space for skill development | 1 (4.8%) |
| **DIVERSITY & PRESENTATION-SPECIFIC LEARNING** |  | **6 (28.6%)** |
| Diverse patient presentations | Exposure to varied clinical presentations | 4 (19.0%) |
| Different presenting problems | Practice with multiple diagnostic categories | 3 (14.3%) |
| Challenging scenarios | Opportunity to practice with difficult cases | 3 (14.3%) |
| Presentation-specific competences | Developing skills tailored to specific presentations | 2 (9.5%) |
| Culturally sensitive practice | Practice with diverse cultural backgrounds (e.g., religion) | 1 (4.8%) |
| **REFLECTION & SELF-AWARENESS** |  | **7 (33.3%)** |
| Conversation analysis | Ability to review and analyze practice interactions | 3 (14.3%) |
| Self-awareness of performance | Enhanced understanding of own strengths and weaknesses | 5 (23.8%) |
| Recognition of missed competencies | Identifying specific competencies not demonstrated | 2 (9.5%) |
| Awareness of specific gaps | Understanding particular areas needing development | 2 (9.5%) |
| Thinking before speaking | Increased mindfulness in communication | 1 (4.8%) |
| **NO PERCEIVED IMPACT** |  | **1 (4.8%)** |
| No impact reported | Participant reported no perceived impact | 1 (4.8%) |

Note: Percentages calculated from N=21 total responses (including 1 null response of "None" and 20 substantive responses). Categories are not mutually exclusive; participants could describe impacts across multiple categories.
